# Exploring the landscape of health technology assessment in Iran: perspectives from stakeholders on needs, demand and supply

**DOI:** 10.1186/s12961-023-01097-0

**Published:** 2024-01-15

**Authors:** Aidin Aryankhesal, Meysam Behzadifar, Ahad Bakhtiari, Saeed Shahabi, Samad Azari, Banafshe Darvishi Teli, Aziz Rezapour, Seyed Jafar Ehsanzadeh, Masoud Behzadifar

**Affiliations:** 1 School of Health Sciences, Faculty of Medicine and Health Sciences, University of East Anglia, Norwich, UK; 2 Social Determinants of Health Research Center, Lorestan University of Medical Sciences, Khorramabad, Iran; 3 Health Equity Research Center (HERC), Tehran University of Medical Sciences (TUMS), Tehran, Iran; 4 Health Policy Research Center, Institute of Health, Shiraz University of Medical Sciences, Shiraz, Iran; 5 Hospital Management Research Center, Health Management Research Institute, University of Medical Sciences, Tehran, Iran; 6 Department of Health Economics, School of Health Management and Information Sciences, Iran University of Medical Sciences, Tehran, Iran; 7 Health Management and Economics Research Center, Health Management Research Institute, Iran, University of Medical Sciences, Tehran, Iran; 8 English Language Department, School of Health Management and Information Sciences, Iran University of Medical Sciences, Tehran, Iran

**Keywords:** Capacity building, Decision-making, Health technology assessment, Health policy, Iran, Resource allocation

## Abstract

**Background:**

The evaluation of health technologies plays a crucial role in the allocation of resources and the promotion of equitable healthcare access, known as health technology assessment (HTA). This study focuses on Iran’s efforts to integrate HTA and aims to gain insights into stakeholder perspectives regarding capacity needs, demand and implementation.

**Methods:**

In this study, we employed the HTA introduction status analysis questionnaire developed by the International Decision Support Initiative (iDSI), which has been utilized in various countries. The questionnaire consisted of 12 questions divided into three sections: HTA need, demand and supply. To identify key informants, we conducted a literature review and consulted with the Ministry of Health and Medical Education (MOHME), as well we experts in policy-making, health service provision and HTA. We selected stakeholders who held decision-making positions in the healthcare domain. A modified Persian version of the questionnaire was administered online from September 2022 to January 2023 and was pretested for clarity. The analysis of the collected data involved quantitative methods for descriptive analysis and qualitative methods for thematic analysis.

**Results:**

In this study, a total of 103 questionnaires were distributed, resulting in a favourable response rate of 61% from 63 participants, of whom 68% identified as male. The participants, when assessing the needs of HTA, rated allocative efficiency as the highest priority, with a mean rating of 8.53, thereby highlighting its crucial role in optimizing resource allocation. Furthermore, healthcare quality, with a mean rating of 8.17, and transparent decision-making, with a mean rating of 7.92, were highly valued for their impact on treatment outcomes and accountability. The importance of budget control (mean rating 7.58) and equity (mean rating 7.25) were also acknowledged, as they contribute to maintaining sustainability and promoting social justice. In terms of HTA demand, safety concerns were identified as the top priority, closely followed by effectiveness and cost-effectiveness, with an expanded perspective on the economy. However, limited access to local data was reported, which arose from various factors including data collection practices, system fragmentation and privacy concerns. The priorities of HTA users encompassed coverage, payment reform, benefits design, guidelines, service delivery and technology registration. Evidence generation involved the participation of medical universities, research centres and government bodies, albeit with ongoing challenges in research quality, data access and funding. The study highlights government support and medical education as notable strengths in this context.

**Conclusions:**

This study provides a comprehensive evaluation of Iran’s HTA landscape, considering its capacity, demand and implementation aspects. It underlines the vital role of HTA in optimizing resources, improving healthcare quality and promoting equity. The study also sheds light on the strengths of evidence generation in the country, while simultaneously identifying challenges related to data access and system fragmentation. In terms of policy priorities, evidence-based decision-making emerges as crucial for enhancing healthcare access and integrating technology. The study stresses the need for evidence-based practices, a robust HTA infrastructure and collaboration among stakeholders to achieve better healthcare outcomes in Iran.

**Supplementary Information:**

The online version contains supplementary material available at 10.1186/s12961-023-01097-0.

## Introduction

Health technology assessment (HTA) encompasses a comprehensive and multidisciplinary process aimed at evaluating the various dimensions of a health technology’s value, which may include aspects such as clinical, economic, social and ethical considerations [1]. The primary objective of HTA is to furnish decision-makers with evidence-based information to facilitate informed healthcare policies, clinical practices and funding choices. Given its pivotal role in evidence generation, HTA plays a crucial role in assessing the benefits, risks and cost-effectiveness associated with novel health technologies [2]. This process is predicated upon an exhaustive appraisal of available evidence, encompassing clinical trials, systematic reviews, observational studies and economic evaluations [3]. Furthermore, HTA takes into account the societal and ethical implications of the technology, including its impact on patient outcomes, quality of life and resource allocation [4]. Particularly in the context of healthcare, where resources are often finite and the implementation of new technologies can prove financially burdensome, HTA assumes heightened significance [5]. By undertaking a rigorous and systematic evaluation of available evidence, HTA serves to ensure the efficient and effective allocation of healthcare resources. Additionally, HTA promotes transparency and accountability in decision-making processes, fortified by the provision of a clear and evidence-based rationale for funding decisions [6].

In addition, universal health coverage (UHC) strives towards ensuring equitable access to comprehensive healthcare services, encompassing prevention, promotion, treatment, rehabilitation and palliation without imposing financial burdens on individuals and communities [7]. In this regard, HTA serves as a vital tool in attaining UHC by evaluating the clinical and economic value of health technologies, thereby aiding informed decision-making by policy-makers concerning the allocation of limited healthcare resources [8]. Utilizing HTA can also contribute to achieving UHC by guaranteeing patients’ access to the most efficacious and cost-effective health technologies, irrespective of their financial capabilities [9]. Moreover, by prioritizing resource allocation and promoting innovation, HTA can facilitate the sustainability of healthcare systems and the provision of high-quality care to all individuals [10].

### Health system in Iran

Iran boasts an extensive public healthcare system that facilitates essential health services to its population [11]. The government operates public hospitals and clinics, often subsidizing services to enhance affordability for the general populace [12]. The role of the private sector is also noteworthy in Iran’s healthcare system, with private hospitals, clinics and medical practitioners offering a diverse range of healthcare services [13]. Private healthcare providers mainly cater to individuals seeking specialized or personalized care and may offer services at varying price points. Iran possesses a domestically developed medical equipment manufacturing industry, generating a plethora of devices and instruments [14]. The procurement process for medical equipment may involve acquiring them from local manufacturers or importing them from international suppliers. Importation and distribution of medical equipment typically necessitate adherence to government regulations and procurement approvals [15]. Iran takes pride in its well-established pharmaceutical industry, responsible for producing an extensive range of medications. The process of acquiring drugs within Iran encompasses a blend of domestic production and importation [16]. While domestically manufactured medications are commonplace, more specialized or patented drugs might be imported. The Ministry of Health and Medical Education (MOHME) shoulders the responsibility of regulating and supervising the pharmaceutical sector, overseeing drug approval and importation [17].

### Current status of HTA in Iran

HTA-related activities were initiated in 2007 with the establishment of a secretariat within the Ministry of Higher Education (MOHE). This initial phase involved the introduction of HTA through workshops and its integration into the agenda. In early 2010, the HTA administration underwent restructuring under the supervision of the Vice of Treatment and the Office of Technology Assessment, Standardization, and Health Tariffs. This restructuring aimed to improve operations and establish a new framework. As the institutions engaged in HTA expanded their activities and individuals received training abroad before returning to Iran, policy-makers in the healthcare sector recognized the importance of cultivating a skilled HTA workforce. Consequently, a Master’s program focused on HTA was established. Currently, four medical science universities have taken the responsibility of educating students in this field. Considering the significant impact of policies related to drugs and medical equipment in Iran, dedicated HTA units were created within both the Food and Drug Organization and the University of Medical Sciences.

In recent years, the healthcare system of Iran has endeavoured to establish mechanisms for incorporating HTA into evidence-based decision-making. Various initiatives have been undertaken towards this end [18, 19]. The objective of this study is to assess the current state of Iran’s healthcare system in terms of the requisite demand, need and supply of HTA services. The findings of this study are expected to offer valuable insights into HTA policies in Iran, thereby aiding policy-makers and decision-makers in the health sector with the successful implementation of UHC.

## Methods

### Ethics declarations

The study was approved by the ethical committee at Lorestan University of Medical Sciences (IR.LUMS.REC.1399.112). All methods were performed in accordance with the relevant guidelines and regulations. The study has also been performed in accordance with the Declaration of Helsinki.

### Instrument of data collection

For this study, the HTA introduction status analysis questionnaire developed by the International Decision Support Initiative (iDSI) at the national level was utilized. This questionnaire has been previously employed in multiple countries, including India, Uganda, Nigeria and various sub-Saharan African nations [22–25]. The questionnaire itself consists of three sections: HTA need, demand and supply encompass a total of 12 questions. The need section encompasses five questions, the demand section consists of two questions and the supply section includes five questions (see Additional file 1 of this paper for the questionnaire).

### Selection of participants and inclusion criteria

The identification of key informants involved an extensive process, which entailed conducting a meticulous literature review and consulting with the MOHME, as well as experts who had experience in policy-making, health service provision, and HTA. A comprehensive search was conducted across various databases, encompassing both national and international sources. The national databases included the Scientific Information Database (SID) (https://www.sid.ir/), MagIran (https://www.magiran.com/), and Elmnet (https://elmnet.ir/), while the international databases comprised PubMed, Scopus, Embase and Web of Science from January 2007 to August 2022. Moreover, official documents, reports and pertinent news related to HTA in Iran were thoroughly examined. Drawing from this accumulated information, a roster of stakeholders was compiled, and subsequently, key experts were selected. The criteria for selecting these key informants were based on their specialized expertise, experience and active involvement in decision-making, resource allocation and prioritization processes within the healthcare sector, both at the regional and national levels. This encompassed individuals who were affiliated with government institutions, research centres, private sectors, companies and organizations, as well as individuals with a work experience of more than 10 years.

### Data collection

For the purpose of data collection, an online questionnaire was utilized between September 2022 and January 2023. The questionnaire underwent a meticulous process of adaptation to the Persian language, involving the use of a forward–backward translation technique. Initially, two translators translated the questionnaire from English to Persian, which was then followed by a reverse translation to English by another translator. Any discrepancies between the original and back-translated versions were resolved through consensus among the translators and authors. To ensure clarity and a consistent understanding among respondents, a pretest was conducted with a sample of seven individuals who were not key informants within the MOHME. This pretest aimed to evaluate the clarity and comprehensibility of the questions from the perspective of the respondents.

All selected participants received a detailed email explaining the objectives of the study and requesting their willingness to participate. The online questionnaire was developed using the Persian platform accessible at https://porsline.ir/. It consisted of a combination of open-ended and multiple-choice questions, allowing participants to express their opinions through online completion. Furthermore, participants were invited to suggest individuals who could potentially contribute to the study’s objectives, as a collaborative approach was considered integral. Efforts were made to ensure diversity in participant representation, which involved actively engaging individuals from various departments associated with HTA and Iran’s healthcare system.

### Data analysis

The method of descriptive analysis was employed to analyse the quantitative data, while the inductive thematic analysis method was applied for analysing the qualitative data. In addition, we conducted data analysis using R Version 4.2.3 software.

## Results

A total of 103 questionnaires were distributed, garnering responses from 63 participants, which translated to a commendable response rate of 61%. In terms of gender distribution, 68% of the participants identified as male. Further details about the participants’ characteristics and their respective fields of activity are presented in Table 1. The mean age of the participants of the study was 39 ± 12 years, and the average work experience was 13 ± 73 years.

1. The need for HTA

**Table 1:** Characteristics of study participants

Variables	Number	Percentage (%)
Sex		
Male	43	68
Female	20	32
Total	63	100
Type of organization		
Government (ministry and civil service)	16	25
Public organization (including autonomous, research institutions)	21	33
Academic institutions (including autonomous public institutes for higher education)	24	38
Other (including private sector and non-governmental organizations)	2	4
Total	63	100
Level		
National	35	56
State	12	19
Both or other	16	25
Total	63	100
Perceived role of own organization in HTA		
Generator	46	73
User	7	11
Both or other	10	16
Total	63	100
Type of organization		
Office within the Ministry of Health	12	20
Office within the Ministry of Finance	6	9
Other government authority	8	12
Regulatory authority	5	7
Health insurance (social or state-funded)	7	11
Health insurance (private)	4	6
Research institute	13	20
Private sector provider	6	9
NGOs	1	3
Others	1	3
Total	63	100

Within Iran’s health system, participants placed particular emphasis on distinct facets of HTA, as indicated by the mean scores out of 10 (see Table 2**)**. Notably, allocative efficiency attained the highest mean score of 8.53. This underscores the pivotal role of optimizing resource allocation to attain optimal health outcomes. Additionally, this approach serves to mitigate health inequalities. Further, enhancing the quality of healthcare received a commendable rating of 8.17. This shows its pivotal role in bolstering treatment efficacy, improving patient experiences and elevating overall health statuses.

**Table 2:** Rating of importance of attributes of HTA

Policy attributes	Mean	Standard deviation
Allocative efficiency	8.53	2.19
Improving quality of healthcare	8.17	2.31
Transparency in decision-making	7.92	2.58
Budget control	7.58	2.03
Equity	7.25	2.82

Transparent decision-making, with a rating of 7.92, was highlighted for its ability to build trust and accountability, promote fairness and facilitate evaluation and feedback. Budget control, rated at 7.58, was recognized as essential for financial sustainability, efficient resource allocation, accountability, and managing healthcare costs. Equity, with a rating of 7.25, was emphasized as a matter of social justice, aiming for equal access to healthcare services and reducing health disparities.

Participant rankings, ranging from 1 to 6, were employed to discern the relative significance of various policy areas where HTA was considered in Iran. The mean scores for these policy areas were then prioritized in the subsequent order, as presented in Table 3.

**Table 3:** Rating of the policy areas

Policy area	Mean	Standard deviation
Coverage or reimbursement of individual health technologies	4.82	1.73
Provider payment reform or pay for performance schemes	4.58	1.39
Informing design of basic package of health benefits	4.21	1.62
Production of clinical guidelines or disease management pathways	4.19	1.45
Health service delivery design	3.97	1.81
Registration of health technologies	3.88	1.42

Regarding the coverage or reimbursement of individual health technologies, participants emphasized the importance of ensuring access and affordability for essential health technologies such as drugs, medical devices and treatments. They recognized the need for effective coverage and reimbursement mechanisms to facilitate access to these technologies. In terms of provider payment reform or pay-for-performance schemes, participants highlighted the significance of incentivizing healthcare providers on the basis of performance and quality of care. They acknowledged that implementing such schemes can encourage healthcare providers to deliver high-quality care and improve health outcomes. Participants also emphasized the value of HTA in informing the design of the basic health benefits package. They recognized the importance of using HTA to identify and prioritize cost-effective interventions and treatments for inclusion in the basic benefits package. The respondents recognized the importance of these guidelines and pathways in guiding healthcare providers to deliver high-quality and consistent care across different healthcare settings. For health service delivery design, participants mentioned improving coordination of care, patient-centredness, and the integration of services as key considerations.

Regarding health technology registration, participants believed that it plays a vital role in ensuring patient safety, promoting quality assurance and building public trust. They recognized the importance of legal and ethical compliance, monitoring and surveillance, and health system planning facilitated by health technology registration. The participants also highlighted the importance of various policy areas in healthcare, including coverage and reimbursement, provider payment reform, HTA-informed benefits package design, production of clinical guidelines, health service delivery design, and health technology registration.

The participants in Iran ranked certain technologies in order of preference, indicating the areas where these technologies can be utilized beyond the HTA (Table 4). The participants prioritized medicines as the top concern, highlighting the crucial role medications play in the healthcare ecosystem. The participants also acknowledged the importance of medication in enhancing overall health outcomes and reducing the burden of diseases on a broader scale. Vaccines assume a critical role in preventing the dissemination of infectious diseases and safeguarding individuals and communities against illnesses that can be averted through vaccination. The ranking underlines the significance of immunization and its contribution to public health promotion. Participants expressed the significance of medical devices and diagnostics within the realm of healthcare. In the case of other interventions, such as surgical procedures, this category encompasses a broad spectrum of interventions beyond pharmaceuticals, including surgical procedures and other medical interventions. Participants recognized the value of these interventions in addressing specific health conditions and providing necessary medical treatments. In addition, participants acknowledged the importance of screening programs aimed at the early detection of diseases and subsequent referral to appropriate healthcare services. Service delivery initiatives or incentives aspire to enhance healthcare delivery and improve patient experiences through innovative models, enhanced accessibility, and incentivized care practices. Participants ranked this category lower, suggesting that while important, it may not be their primary focus compared with other technologies.

**Table 4:** Types of health technology in which the output is from a HTA process

Type of health technology assessment	Mean	Standard deviation
Medicines	4.92	1.72
Public health programs or initiatives	4.58	1.56
Vaccines	4.32	1.09
Medical devices/diagnostics	4.16	1.98
Other intervention (e.g. surgical procedures)	3.97	1.62
Screening and referral programs	3.82	1.19
Service delivery initiatives or incentives	3.18	1.58

### Priority health or healthcare issues

Considering economic problems, sanctions, increase in the elderly population and demand for new technologies in Iran, participants in the study identified two priority health and treatment issues for Iran’s health system.

1. Affordable access to healthcare services: Given the economic challenges and sanctions affecting Iran, it is crucial to prioritize affordable access to healthcare services. This involves ensuring that individuals, including those with limited financial resources, have financial access to healthcare. By addressing affordability, we can reduce financial barriers and ensure that healthcare is accessible to the entire population.
2. Integration of health technologies and innovation: With the increase in the elderly population and the demand for new technologies, it is necessary to prioritize the integration of health technologies and innovation in the healthcare system. Technology can play a vital role in improving healthcare delivery, enhancing efficiency and expanding access to healthcare services, particularly in remote or underserved areas. By embracing new technologies, we can optimize resource allocation, improve the overall quality of care and better meet the healthcare needs of the population, including the elderly.

2. Demand for HTA

On the basis of the participants’ responses, the three potential users of HTA outputs in Iran include the following organizations (Table 5). Participants’ level of interest in the types of HTA outputs is summarized in Table 6.

**Table 5:** Potential users of HTA outputs in Iran and their funding sources and evidence requirements

Number	Name of organization	Type of organization	Funding sources	Required evidence
1	Ministry of Health and Medical Education (MOHME)	Government	Government budget, public funds	The MOHME relies on HTA outputs to inform healthcare policy and decision-making. They require evidence on the clinical effectiveness, cost-effectiveness, safety, and feasibility of health technologies to make informed decisions regarding their inclusion, reimbursement or utilization within the healthcare system
2	Ministry of Cooperation, Labor and Social Welfare (MCLSW)	Government	Government budget, public funds	The MCLSW may utilize HTA outputs to inform policies related to social welfare and healthcare services for specific populations, such as the elderly and vulnerable groups. They require evidence on the impact of health technologies on social welfare, cost-effectiveness and benefits to support decision-making regarding resource allocation and service provision
3	Government and private insurance organizations	Government and private sector	Government funds, insurance premiums, private funding	Government and private insurance organizations may utilize HTA outputs to inform coverage decisions and reimbursement policies. They require evidence on the clinical and cost-effectiveness of health technologies to determine coverage eligibility, reimbursement rates and utilization management strategies. This helps in ensuring that insurance resources are allocated appropriately on the basis of the value and impact of the technologies

**Table 6:** Level of respondents’ interest in the use of different types of HTA outputs

Use of health technology assessment output	Mean level of interest	Standard deviation
Safety	9.61	2.18
Efficacy	9.29	2.14
Cost-effectiveness	8.35	2.13
Economics (e.g. value for money, costs, budget impact)	8.17	2.61
Social/ethical considerations (e.g. equity, solidarity)	7.84	2.08
Other	7.21	2.93

In relation to safety, participants have ranked it as their primary concern due to the potential risks and consequences associated with healthcare interventions. With regard to effectiveness, participants have emphasized the significance of healthcare technologies being efficacious, as this directly influences patient outcomes. In terms of cost-effectiveness, the findings indicate that participants understand the importance of efficient resource allocation and maximizing the value of healthcare investments. In regard to the economy, participants’ ratings reveal that they considered economic factors beyond affordability. These factors encompassed considerations of sustainability and the availability of healthcare resources. Participants recognized the importance of evaluating the financial implications of implementing health technologies. Further, the relatively low ranking of social/ethical considerations can be attributed to several factors. Although participants acknowledged its significance, they prioritized other aspects, such as safety and effectiveness, due to the demands placed on the healthcare system by the people in Iran. These aspects were perceived as more pressing concerns in terms of priority. Lastly, concerning the three organizations introduced by the participants, the ranking points for the mentioned items are outlined in Table 7.

3. Supply of HTA

**Table 7:** The ranking points for three organizations introduced by the participants

Organizations	Level (0–10)	Types of evidence required				
		Safety	Efficacy	Effectiveness	Economics (e.g. value for money, costs, budget impact)	Social/ethical concerns (e.g. equity, solidarity)
1. MOHME		9	8	9	7	6
Explanation	As a key governmental body responsible for healthcare policy- and decision-making, MOHME places a high level of importance on safety, efficacy and effectiveness evidence. They require robust evidence on the clinical aspects of healthcare technologies. Additionally, they have a moderate level of interest in economic evidence to ensure value for money. Social/ethical concerns are also considered at a moderate level, particularly regarding equity and correlation with healthcare interventions					
2. MCLSW		7	7	6	6	7
Explanation	MCLSW, responsible for social welfare and targeted healthcare services, requires evidence on safety, efficacy and effectiveness at a moderate level. They consider the clinical aspects of healthcare technologies to ensure the wellbeing of specific populations. Additionally, they have a moderate interest in economic evidence, including value for money and budget impact. Social/ethical concerns are also considered at a moderate level, focusing on equity and correlation					
3. Government and private insurance organizations		8	8	9	9	7
Explanation	These organizations, responsible for insurance coverage and reimbursement decisions, require a high level of evidence on safety, efficacy, effectiveness and economics. They emphasize comprehensive evidence to ensure the safety and effectiveness of technologies. Economic evidence, including value for money, costs and budget impact, is crucial for making coverage and reimbursement decisions. Social/ethical concerns are also considered, albeit at a moderate level, with a focus on equity considerations					

The participants were asked to state the strengths and weaknesses of their organizations in relation to evidence-based healthcare in Iran. The strengths and weaknesses are mentioned below.

### Strengths

*Research and academic institutions*: Iran has various reputable research and academic institutions dedicated to healthcare.

*Collaboration with international organizations:* Iran’s healthcare organizations often collaborate with international organizations and research institutions.

*Government support:* The Iranian government recognizes the importance of evidence-based healthcare and has taken steps to support it. They have established policies and initiatives to promote evidence-based practices and research in healthcare organizations.

*National research networks:* Iran has established national research networks, which facilitate the implementation of evidence-based healthcare.

*Health information systems:* Iran has made significant progress in developing health information systems. These systems help collect, analyse and disseminate health-related data, including evidence-based research findings.

*Emphasis on continuing medical education:* Continuing medical education is a priority in Iran’s healthcare system. Healthcare professionals are encouraged to stay updated with the latest evidence-based practices through continuous learning and professional development programs.

*Use of clinical practice guidelines:* Iran has developed and implemented clinical practice guidelines in various healthcare specialities. These guidelines are based on rigorous evidence and provide healthcare practitioners with standardized recommendations for diagnosis, treatment and management of different conditions.

*Public health initiatives:* Iran has implemented various public health initiatives on the basis of evidence. These initiatives target key health issues, such as preventive measures, health promotion and disease control.

### Weaknesses

*Limited access to up-to-date research findings:* Healthcare organizations face challenges in accessing international journals, databases and research resources, which can hinder their ability to stay updated with the most current evidence.

*Research funding constraints:* Insufficient financial resources may restrict the scope and scale of research projects, making it difficult to generate robust evidence to support healthcare practices.

*Research infrastructure challenges:* Limited access to state-of-the-art laboratories, advanced equipment and research facilities can hinder the execution of rigorous studies and evidence generation.

*Variability in research quality:* Variability in research quality may arise due to factors such as limited resources, inadequate training or lack of standardized research methodologies. This can affect the reliability and generalizability of the evidence produced.

*Limited implementation of evidence:* Despite the emphasis on evidence-based healthcare, organizations usually face barriers such as resistance to changes, lack of awareness among healthcare providers or inadequate support systems for translating evidence into practice.

*Fragmented healthcare system:* Iran’s healthcare system is characterized by a fragmented structure, with multiple stakeholders involved in the delivery of care. This fragmentation can lead to challenges in the coordination and dissemination of evidence-based practices across different healthcare organizations and settings.

*Data quality and standardization:* Variability in data collection, documentation practices and record-keeping can affect the reliability and comparability of research findings and evidence-based guidelines. The participants highlighted the availability of local data as a key challenge in conducting HTA in Iran (Table 8).

**Table 8:** Availability of four sources of data listed

Source data	Available *N* (%)	Not available *N* (%)	Available with limitations *N* (%)	Total *N* (%)
Activity of hospitals	31	8	24	63 (100)
Health outcomes	29	18	16	63 (100)
Service delivery	19	35	9	63 (100)
Pharmaceutical usage and pricing	5	42	16	63 (100)

The participants raised multiple concerns pertaining to data collection and documentation practices within Iranian healthcare organizations, thereby resulting in restricted access to local data. Insufficient or incomplete recording of healthcare activities, health outcomes and service delivery information, as well as drug utilization and pricing data, can hinder the availability of comprehensive and reliable local data for HTA. In the context of Iran’s healthcare system, data access and sharing among different stakeholders impose limitations on local data for HTA. Participants identified challenges such as data privacy concerns, the lack of standardized data sharing protocols and inadequate data infrastructure as hindrances to the efficient exchange of data. Moreover, participants reported that inadequate research capacity and funding are additional factors that may obstruct the generation of local data for HTA. Limited resources for research studies, clinical trials and data collection designs may lead to a dearth of comprehensive and up-to-date local data, thus compromising the informative value of HTA processes. The participants also noted that variability in data quality and the absence of standardized data collection methods may further undermine the availability of reliable local data.

### Organizations involved in the supply or generate evidence in Iran

In Iran, various organizations play a role in supplying or generating evidence to support health policy decisions. These organizations include:

1. Medical universities: Medical universities in Iran often engage in research activities and contribute to the generation of evidence. They conduct studies, clinical trials and research projects to gather data and generate evidence relevant to health policy decisions.
2. Research centres: Research centres in Iran play a significant role in generating evidence. These centres focus on specific areas of research, such as public health, epidemiology or medical sciences. They conduct studies, collect data and analyse information to generate evidence that can inform health policy decisions.
3. Government institutions: Government institutions in Iran are responsible for health policy development and implementation. They often rely on evidence to make informed decisions. These institutions may conduct their research or rely on evidence generated by medical universities and research centres to support health policy decisions.

Overall, the identified organizations in Iran, including medical universities, research centres and government institutions, have a role in both supplying and generating evidence. They contribute to the evidence base by conducting research, collecting data, analysing information and providing evidence to support health policy decisions.

### HTA infrastructure in Iran’s health system

In Iran, efforts have been made to establish and strengthen the infrastructure for HTA, as presented in Table 9.

**Table 9:** Initiatives for HTA Infrastructure in Iran

Activities	Description
Appropriate research centres	Establishment of research centres focused on conducting HTA studies
HTA unit	Formation of a dedicated unit within the Ministry of Health for overseeing HTA activities
Capacity building	Initiatives to enhance knowledge and skills of professionals involved in HTA
Familiarization workshops on HTA	Workshops to create awareness and understanding of HTA principles and practices
Manpower training in universities	Training programs in universities to educate individuals in HTA methodologies and processes
Development of local data network	Efforts to develop and strengthen a local data network for reliable and relevant HTA data
Development of guidelines	Creation of standardized guidelines for conducting HTA studies
Collaboration with other stakeholders	Strengthening collaboration with various stakeholders to generate and utilize evidence effectively
Development and policy-making for HTA utilization	Policy development to promote the use of HTA in healthcare decision-making

### Training needs for generators to improve HTA capacity

Participants provided a comprehensive list of training needs for capacity building in HTA for evidence generators. Training needs are rated on a scale of 1 to 10 (Table 10).

**Table 10:** Training needs for generators to improve HTA capacity

Training program	Mean	Standard deviation
Introduction and application of HTA	7.82	1.84
Topic selection process for HTA	7.37	1.29
Health economic evaluation & economic modelling	7.29	1.47
Systematic reviews	7.15	1.04
Meta-analysis	6.97	1.25
Costing healthcare	6.12	1.83
Measuring health outcomes	5.94	1.91
Other	5.62	1.02

Participants emphasized the significance of introducing and applying HTA, as it establishes the groundwork and facilitates a fundamental comprehension of HTA principles and practices. In the process of selecting topics for HTA studies, participants stressed the importance of choosing appropriate subjects to ensure that resources are allocated to areas with the greatest impact and need. The inclusion of health economic evaluation and economic modelling was considered highly valuable, given the growing importance of cost-effectiveness analysis and economic modelling in HTA. These methodologies assist decision-makers in evaluating the value of health technologies relative to their costs. Systematic reviews and meta-analyses play a crucial role in synthesizing and analysing available evidence, providing a robust foundation for HTA assessments. The consideration of healthcare cost is essential for understanding and estimating the financial implications associated with implementing health technologies, aiding in decision-making regarding resource allocation. Lastly, the measurement of health outcomes is vital for evaluating the effects of health technologies on patient outcomes and overall population health.

### Training needs for users to improve HTA capacity

The participants rated the training needed for users in Iran to improve HTA capacity from 1 to 5. The ranking of each training, subject and their scope are presented in Table 11.

**Table 11:** Training needs for improving HTA capacity among users in Iran

Training needs	Mean	Standard deviation	Subjects	Scope
Introduction and Application of HTA	4.87	1.27	Basic principles and concepts of HTA	Understanding the fundamentals of HTA methodologies and their practical application in decision-making
Topic selection process for HTA	4.62	1.93	Criteria and methods for selecting HTA topics	Learning how to engage stakeholders and prioritize topics for HTA
Institutional processes for HTA	4.11	1.61	Organizational structures and governance models for HTA	Understanding the role and responsibilities of HTA agencies/committees within healthcare systems
Overview of health economics	4.02	1.15	Introduction to health economics and its relevance to HTA	Gaining knowledge of economic evaluation methods and interpreting economic evidence in HTA
Using evidence for policy-making	3.94	1.72	Evidence-based policy-making and critical appraisal of studies	Learning strategies for incorporating evidence into policy decisions and evaluating research studies
Other	3.71	1.05	Systematic literature review methods and ethical considerations in HTA	Understanding HTA data collection and analysis, health outcomes assessment and decision modelling

## Discussion

This study represents the inaugural attempt aimed at delineating the current trajectory of HTA in Iran. The Iranian health system adopts a hierarchical approach to decision-making and policy formulation. However, the prompt consideration of regional health matters remains insufficient, necessitating the incorporation of the nation’s cultural and geographical diversity during the implementation of diverse technologies.

The findings of this study yield valuable insights into the pivotal role of HTA in facilitating well-informed decision-making, thereby contributing to the attainment of UHC in Iran. These results demonstrate the significance of addressing training requirements, policy domains, stakeholder interests and the availability of local data, thus elucidating the crucial determinants influencing the capacity for HTA and evidence-based healthcare within the country.

Participants in this investigation emphasized the fundamental significance of HTA in healthcare technology coverage and reimbursement policy. Over the past decade, Iran’s healthcare system has witnessed an escalated demand for HTA while grappling with several financial constraints caused by various factors such as sanctions, the coronavirus disease 2019 (COVID-19) outbreak and ageing population. The contemporary landscape of healthcare is marked by a plethora of innovative technologies, yet a substantial number of these advancements find themselves in a state of neglect, unsupported by insurance plans. Both public and private insurance entities exhibit a reluctance to embrace these novel technologies, creating a formidable barrier to their widespread accessibility and adoption.

One of the primary culprits behind this reluctance is the exorbitant cost associated with certain health technologies, a significant impediment, particularly for individuals with limited financial means [26]. This paper emphasizes the pivotal role of HTA within the broader framework. In the evaluation of coverage and reimbursement, HTA emerges as a linchpin, offering a systematic approach to gauging the value and affordability of these health technologies [27]. By scrutinizing the potential benefits and costs linked to providing coverage and reimbursement for these technologies, healthcare policy-makers and payers are empowered to make judicious decisions. These decisions extend beyond mere financial considerations; they encompass the broader impact on patient outcomes, healthcare delivery and the overall efficacy of the healthcare system [28]. The integration of HTA into insurance plans emerges as a strategic imperative, resonating with a body of research that underscores its significance and potential [22, 25, 29].

Against the backdrop of financial constraints within the Iranian health system, policy-makers find themselves grappling with heightened concerns, exacerbated by the substantial expenditures attributable to the COVID-19 pandemic and disease management [19]. In response to these challenges, study participants underscored the imperative for judicious allocation of financial resources and the advancement of evidence-based healthcare services [19]. Addressing these concerns, Dang et al. draw attention to the pivotal role of HTA as a strategic tool for bolstering the efficiency of the health system. This recommendation is contextualized within the broader landscape of escalating healthcare costs, the unique demographic profile of Iran and the swift pace of technological advancements[7].

The examination of various types of technology within the study revealed a discernible preference among participants for advancements in medicine, health interventions and vaccines. Strikingly, this inclination mirrors the outcomes of a survey report by the WHO. The WHO report underscores that in many low-income countries, there exists a consensus regarding the indispensability of medicine and health interventions as pivotal technologies for HTA. This collective sentiment implies that a substantial portion of healthcare costs in these regions is intricately linked to these specific technologies. Moreover, the prevalence of diverse diseases, both in low-income and high-income countries, appears to exert a profound influence on the preferences for specific technologies within their respective healthcare systems [30, 31].

This study provides invaluable information with Iran’s MOHME, MCLSW and government and private insurance organizations as the primary users of HTA, which is consistent with findings from precursory studies [1, 22, 25] and the WHO report. As custodians of health within their respective countries, these organizations bear the responsibility of providing services through the utilization of various technologies and ensuring their financial coverage.

Among the limitations identified by the participants, the lack of appropriate local and national data was particularly emphasized. They considered the existing data to be insufficient and limited [32]. Notably, there is a pressing need for investment in the establishment and enhancement of data systems for health research to support HTA analysis in Iran. However, the application of evidence based on HTA requires both financial resources and political support, both of which are crucial for its development [33].

To garner political support, it is crucial to focus on capacity building and raising awareness among policy-makers regarding the potential of HTA [34]. Furthermore, there is a certain need for skilled human resources to expand HTA research in Iran, and although some efforts have been made in recent years to develop relevant training, such as the establishment of HTA courses in medical universities, there remains room for improvement. Regrettably, the MOHME has not earnestly incorporated the utilization of trained human resources in the field of HTA into its agenda, which has resulted in numerous policy decisions being made without their involvement and the necessary evidence preparation [35].

In Iran’s healthcare system, decision-making primarily resides within the MOHME. However, leveraging the expertise of trained human resources across different regions can significantly contribute to the preparation of localized evidence and subsequently serve as a valuable bridge for national-level decision-making. Establishing effective channels of communication is vital in bolstering Iran’s capacity for HTA research.

### Limitation

This study has some limitations that warrant acknowledgment. Firstly, a majority of the study’s participants were affiliated with government organizations or research institutes, potentially limiting the representativeness of non-governmental organizations or the private sector. Additionally, the study may have been subject to selection bias, stemming from organizations’ willingness to participate or the identification of relevant entities. Another limitation pertains to missing data or complications in the analysis, potentially impacting the generalizability of the findings.

## Conclusions

Although the need for the establishment of HTA is generally acknowledged by authorities and health sector policy-makers in the context of achieving UHC, it is imperative for them to place HTA as a priority over policies influenced by specific stakeholders for their individual gains. HTA should be employed as a means to assess new health technologies. To secure political support and commitment towards HTA, it is essential to enhance the understanding of HTA fundamentals among policy-makers and healthcare managers.

### Supplementary Information

**Additional file 1.** Questionnaire.

## Data Availability

All data generated or analysed during this study are included in this published article.

## Abbreviations

HTA: Health technology assessment
iDSI: International decision support initiative
MOHME: Ministry of Health and Medical Education
UHC: Universal health coverage
SID: Scientific information database
WHO: World Health Organization
