# Supplementary material for: Exploring the landscape of health technology assessment in Iran: perspectives from stakeholders on needs, demand and supply
Source: Health Res Policy Syst. 2024 Jan 15;22:11. doi: 10.1186/s12961-023-01097-0 (PMC10789076; doi:10.1186/s12961-023-01097-0)
Supplement: Supplementary file 1 — Additional file 1. Questionnaire. [file 12961_2023_1097_MOESM1_ESM.pdf]

Lorestan University of Medical Sciences

Situation analysis of health technology assessment (HTA) introduction at national level

Dear Participant,

Hello,

This questionnaire has been designed and made available to you with the aim of gathering your views regarding the three items: needs, demand, and supply for HTA in Iran's health system. Thank you for participating in this research. We request you to express your views according to the questions raised. Your information will remain confidential with the researchers.

Thank you for your cooperation,

Research team

Sex: Male ☐ Female ☐

Age: …………….. years

Type of organization:

- Government (Ministry and Civil Service) ☐
- Public Organization (including autonomous, research institutions) ☐
- Academic Institutions (including autonomous public institutes for higher education) ☐
- Other (including private sector and non-governmental organizations) ☐

Level: National ☐ State ☐ Both or other ☐

Perceived role of own organization in HTA: Generator ☐ User ☐ Both or other ☐

Type of your organization:

Office within the Ministry of Health ☐ Office within the Ministry of Finance ☐

Other government authority ☐ Regulatory authority ☐

Health insurance (social or state-funded) ☐ Health insurance (private) ☐

Research institute ☐ Private sector provider ☐

NGOs ☐ Others ☐

Questionnaire

Part I: Priority setting of need for HTA

1. **There are many attributes of health technology assessment that makes it a useful policy device. For each of the attributes listed under a. to e. below, please rate their importance to health policy in your Iran by circling a number on the scale where 0 is not important and 10 is very important. Please provide reasons for your answers where possible:**

a. Ensuring that the money and resources available for health are distributed in the best way to achieve maximum benefits from the money available (allocative efficiency)

0	1	2	3	4	5	6	7	8	9	10
**Not important**										**Very important**

b. Transparency in decision making

0	1	2	3	4	5	6	7	8	9	10
**Not important**										**Very important**

c. Budget control

0	1	2	3	4	5	6	7	8	9	10
**Not important**										**Very important**

d. Equity

0	1	2	3	4	5	6	7	8	9	10
**Not important**										**Very important**

e. Improving quality of health care

0	1	2	3	4	5	6	7	8	9	10
**Not important**										**Very important**

2. HTA can be used in many different health policy areas to improve the evidence base used in the decision-making process. Please choose up to three of the following policy areas, in which the output from a HTA process is urgently needed in Iran.
If you choose more than one policy area, please rank in order of importance where 1 is the most important. Please also provide reasons for your choice.

☐ Registration of health technologies, because:

……………………………………………………………………..……………………………………………………………………….....

☐ Coverage or reimbursement of individual health technologies, because:

……………………………………………………………………..……………………………………………………………………….....

☐ Production of clinical guidelines or disease management pathways, because:

……………………………………………………………………..……………………………………………………………………….....

☐ Informing design of basic package of health benefits, because:

……………………………………………………………………..……………………………………………………………………….....

☐ Health service delivery design, because:

……………………………………………………………………..……………………………………………………………………….....

☐ Provider payment reform or pay for performance schemes, because:

……………………………………………………………………..……………………………………………………………………….....

3. **HTA can be used to assess many different types of health technology. Depending on the situation in Iran, particular health technologies might be more in need of HTA than others. Please choose up to three of the following types of health technology, in which the output from a HTA process is urgently needed in your country.**

<u>If you choose more than one health technology, please rank in order of importance where 1 is most important</u>.

Please also provide reasons for your choice. Reasons might include (but are not limited to) measures such as burden of illness, budget impact, potential for benefit, or patient/clinician advocacy.

☐ Medicines

Reasons……

☐ Vaccines

Reasons………

☐ Medical devices / diagnostics

Reasons…………………………………………………………………………………………………..………

☐ Other intervention (e.g. surgical procedures)

Reasons……

☐ Screening and referral programs

Reasons ………

☐ Public health programs or initiatives

Reasons……

☐ Service delivery initiatives or incentives

Reasons……

4. **Please identify two priority health or health care issues for your institution/department and explain why these issues are important.**

(1) ....

(2) ....

5. **From the priority issues identified in question four, please provide two examples of questions that should be researched considering the provided criteria.**

(1) ……

(2) ……

Part II: Identifying the demands (for evidence)

6. Who are the potential users of HTA outputs in Iran? Please identify not more than three organizations and provide the name and type of organizations, their funding sources, and required evidence in the provided table. If there are related documents available, please note below and provide separately.

Number	Name of organization	Type of organization	Funding sources	Required evidence
1				
2				
3				

7. From the evidence users that you listed in question six, please rate the level of their interest in different types of HTA outputs. In the grey shaded areas, please use a 0-10 scale where 0 is no demand and 10 is high demand. Please also provide explanations and examples.

0	1	2	3	4	5	6	7	8	9	10
No demand										Highly demand

Organizations		Types and level of evidence required				
		Safety	Efficacy	Effectiveness	Economics (e.g. value for money, costs, budget impact)	Social/ethical concerns (e.g. equity, solidarity)
1	Level (0-10)					
	Explanations /examples					
2	Level (0-10)					
	Explanations /examples					
3	Level (0-10)					
	Explanations /examples					

Part III: Exploring the supply (for evidence)

8. **Please identify your organization's strengths as well as weaknesses in relation to evidence generation or use.**

Strengths:

………

………

………

………

………

………

Weaknesses:

………

………

………

………

………

………

9. Availability of local data to inform country-specific decision is a key challenge of HTA. Please indicate the availability of the following data:

Pharmaceutical usage and pricing

☐ Available ☐ Not available

☐ Available with limitations (please identify) ……………………………………………………………………………

………..……………

Activity of hospitals – e.g. how many times is a particular inpatient or outpatient hospital procedure performed per month in an individual hospital, or across regions, or nationally

☐ Available ☐ Not available

☐ Available with limitations (please identify) ……………………………………………………………………………

………..……………

Health outcomes – e.g. what is the average 30 day mortality following admission to a hospital for acute myocardial infarction (heart attack) at an individual hospital, or across regions, or nationally

☐ Available ☐ Not available

☐ Available with limitations (please identify) ……………………………………………………………………………

………..……………

Service delivery – e.g. health professional salaries

☐ Available ☐ Not available

☐ Available with limitations (please identify) ……………………………………………………………………………

………..……………

10. **Please identify the organizations that supply or generate evidence to support health policy decisions in Iran. <u>Please provide names of three leading organizations and contact persons, if possible</u>, and indicate the role of identified organizations whether they supply or generate evidence.**

(1) ………

(2) ………

(3) ………

11. Please provide brief information regarding HTA infrastructure available in Iran? , e.g. guidelines for the methods of health technology assessment or databases. If there are related documents available, please feel free to share.

……………………………………………………………………………………………………

……………………………………………………………………………………………………

……………………………………………………………………………………………………

……………………………………………………………………………………………………

……………………………………………………………………………………………………

……………………………………………………………………………………………………

……………………………………………………………………………………………………

……………………………………………………………………………………………………

……………………………………………………………………………………………………

……………………………………………………………………………………………………

……………………………………………………………………………………………………

12. If any, please identify training needs including subjects and scope to improve HTA capacity of both evidence generators and users.

(1) ....

(2) ....

(3) ....
